# Divergent *MLS1* Promoters Lie on a Fitness Plateau for Gene Expression

**DOI:** 10.1093/molbev/msw010

**Published:** 2016-01-18

**Authors:** Andrew C. Bergen, Gerilyn M. Olsen, Justin C. Fay

**Affiliations:** ^1^Molecular Genetics and Genomics Program, Washington University, St. Louis; ^2^Washington University, St. Louis; ^3^Department of Genetics, Washington University, St. Louis; ^4^Center for Genome Sciences and Systems Biology, Washington University, St. Louis

**Keywords:** yeast, evolution, expression, MLS1, binding sites.

## Abstract

Qualitative patterns of gene activation and repression are often conserved despite an abundance of quantitative variation in expression levels within and between species. A major challenge to interpreting patterns of expression divergence is knowing which changes in gene expression affect fitness. To characterize the fitness effects of gene expression divergence, we placed orthologous promoters from eight yeast species upstream of malate synthase (*MLS1*) in *Saccharomyces cerevisiae*. As expected, we found these promoters varied in their expression level under activated and repressed conditions as well as in their dynamic response following loss of glucose repression. Despite these differences, only a single promoter driving near basal levels of expression caused a detectable loss of fitness. We conclude that the *MLS1* promoter lies on a fitness plateau whereby even large changes in gene expression can be tolerated without a substantial loss of fitness.

## Introduction

Changes in gene regulation are thought to play an important role in evolution (King and Wilson 1975; Wray 2007; Carroll 2008). Although there are many examples of *cis*-regulatory changes underlying diverged phenotypes (Wray 2007; Gaunt and Paul 2012), phenotypes more often map to changes in protein-coding sequences (Hoekstra and Coyne 2007; Stern and Orgogozo 2008; Fay 2013; Martin and Orgogozo 2013). One reason for the fewer number of phenotypes attributable to *cis*-regulatory mutations is the greater difficulty in demonstrating their influence on a phenotype (Stern and Orgogozo 2008). As such, most of our understanding of regulatory evolution is based on gene expression and *cis-*regulatory sequence divergence irrespective of downstream phenotypes.

One general feature of regulatory evolution that has emerged is conservation of qualitative patterns of gene expression despite divergence in the *cis-*regulatory sequences driving expression. Quantitative studies of gene expression levels have shown that there is an abundance of quantitative variation within and between species (Whitehead and Crawford 2006; Gordon and Ruvinsky 2012). Yet, qualitative patterns of activation, repression, and tissue-specific expression are generally conserved across distantly related species (Gasch et al. 2004; Chan et al. 2009). In comparison, studies of *cis*-regulatory sequences have shown that gain and loss of transcription factor binding sites is common (Moses et al. 2006; Doniger and Fay 2007; Kim et al. 2009; Bradley et al. 2010; Schmidt et al. 2010; Yokoyama et al. 2014), and that between distantly related species, *cis*-regulatory sequences often diverge to the extent that the sequences are unalignable (Wratten et al 2006; Hare et al 2008; Venkataram and Fay 2010; Arnold et al. 2014).

The binding site turnover model explains how gene regulation can be conserved while *cis*-regulatory sequences diverge (Ludwig et al. 1998 , 2000; Dermitzakis and Clark 2002; Dermitzakis et al. 2003). Under this model, gain and loss of equivalent binding sites within the same regulatory sequence enables high rates of divergence without changes in gene regulation. The binding site turnover model is supported by striking demonstrations that diverged *cis*-regulatory sequences from distantly related species drive very similar patterns of gene expression when placed in the same genome (Romano and Wray 2003; Ruvinsky and Ruvkun 2003; Markstein et al. 2004; Fisher et al. 2006; Wratten et al. 2006; Hare et al. 2008; Swanson et al. 2011; Arnold et al. 2014). Over long time periods, divergence in *cis*-regulatory sequences may also be facilitated by transcriptional rewiring, whereby different binding sites can be substituted for one another (Tsong et al. 2006; Tuch et al. 2008). However, the decrease in regulatory conservation as *cis*-regulatory sequences are placed into more distantly related genomes (Gordon and Ruvinsky 2012; Barrière and Ruvinsky 2014) implies that there are limits to the compatibility of *cis*-regulatory sequences with distantly related *trans*-environments.

A major barrier to interpreting patterns of gene expression conservation and divergence is that their influence on outward phenotypes or fitness is unknown. Although in some instances patterns of gene expression divergence themselves are indicative of fitness effects, in most cases expression divergence is assumed to be neutral (Fay and Wittkopp 2008). For example, there is evidence that subtle but consistent changes in the expression of genes in the same pathway or biological process influence fitness (Bullard et al. 2010; Fraser *et al.* 2010). A further complication is that fitness may depend not only on expression levels. The temporal or developmental patterns of expression may also influence fitness. For example, by comparing the distribution of mutation effects with naturally occurring polymorphism in the gene *TDH3*, Metzger *et al.* (2015) inferred that there is abundant purifying selection against mutations that increase cell to cell variation in expression levels. Overall, testing whether *cis*-regulatory sequences have diverged in their ability to integrate transcription factors, nucleosome positioning, and core transcriptional machinery into proper expression is challenging. However, the consequences of any meaningful regulatory changes should be reflected in fitness.

The direct effects of gene expression on fitness are not often characterized. Ludwig et al. (2005) found complementation of diverged enhancers, although none of the transgenic constructs rescued wild-type fitness levels. Using an inducible promoter, a fitness plateau was found for *LCB2* gene expression in yeast (Rest et al. 2013). In this case, fitness increased with gene expression levels, but above a certain level no further changes in fitness were observed (Rest et al. 2013). Although there are also many examples of expression changes that underlie phenotypes likely to influence fitness (Hoekstra and Coyne 2007; Stern and Orgogozo 2008), it is difficult to make generalizations about the nature of these expression changes.

Here, we examine the effects of promoter divergence on both gene expression and fitness in yeast using the malate synthase (*MLS1*) promoter. As part of the glyoxylate cycle, *MLS1* is induced in the absence of fermentable carbon and repressed in the presence of glucose (Turcotte et al. 2010). *MLS1* converts acetyl-CoA into malate and is necessary for gluconeogenesis and growth on nonfermentable carbon sources (Hartig et al. 1992). The enzymatic function of Mls1 has been demonstrated to be conserved in *Kluyveromyces lactis* (Georis et al. 2000). Additionally, *MLS1* has a well characterized promoter, where the main transcription factor binding sites and regions necessary for activation and repression have previously been identified (Caspary et al. 1997). Activation of *MLS1* occurs through two Abf1 binding sites, responsible for basal expression levels, and two Cat8 binding sites, responsible for its large increase in expression following depletion of glucose (Caspary et al. 1997). Cat8 binding sites have also been shown to be bound by Sip4 (Roth et al. 2004). The main transcription factors that control *MLS1* expression are conserved across species. Activation of *MLS1* by the transcription factor *CAT8* is conserved in *K. lactis*, a species that split before the whole-genome duplication and a shift in metabolism from respiratory to fermentative growth in the presence of oxygen (Georis et al. 2000). Repression of *MLS1* occurs through a Mig1 site (Caspary et al. 1997). It has been shown that a *MIG1* gene deletion in *Saccharomyces cerevisiae* can be rescued by *MIG1* from *Candida utilis* (Delfin et al. 2001) and *K. lactis* (Cassart et al. 1995), indicating that *MIG1* has conserved its general function as well.

Assays of orthologous *cis*-regulatory sequence function in a single species background have previously been valuable in understanding how they evolve. For example, loss of function can be caused by incompatibility between *cis*-regulatory sequences and *trans*-acting factors (Barrière et al. 2012) or by gain and loss of binding sites within the same *cis*-regulatory sequence (Ludwig et al. 2000). Here, we place orthologous *MLS1* promoters from eight different yeast species into *S*. *cerevisiae* to determine what selective constraints act on this promoter as well as what expression levels and dynamics *S. cerevisiae* requires for *MLS1* function. We expected and found that orthologous promoters caused differences in gene expression levels while maintaining the general pattern of activation and repression. We then used competitive growth assays to show that despite varying expression levels, all but one of the species’ promoters completely rescues competitive fitness in *S. cerevisiae*. Our results demonstrate that most of the diverse configurations of binding sites within the *MLS1* promoter drove expression levels that can be tolerated without substantial fitness effects.

## Results

### High Sequence Divergence with Conservation of Binding Site Content

**Fig. 1.**
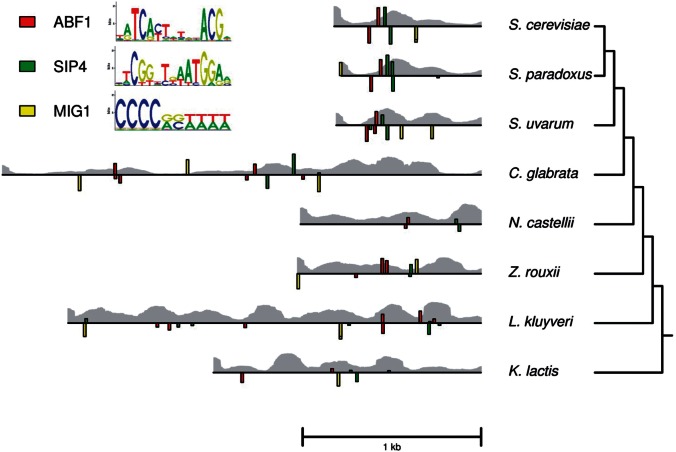
Transcription factor binding and nucleosome occupancy predictions for *MLS1* promoters. The noncoding region upstream of the *MLS1* start codon from eight yeast species, where the heights of colored bars represent the scores of predicted binding sites for Abf1, Sip4, and Mig1 based on PWMs from MacIsaac et al. (2006). Bars above each line represent sites on the forward strand and those below represent sites on the reverse strand. The probability of nucleosome occupancy at each base pair along the promoters is represented by the height of the gray bars in the background. Phylogenetic relations are based on Scannell et al. (2006).

To characterize sequence divergence in the *MLS1* promoter, we examined the noncoding sequences between *MLS1* and the codon region of the upstream gene in eight yeast species. Similar to genome-wide patterns of promoter evolution in yeast (Venkataram and Fay 2010), the *MLS1* promoter exhibits the following: 1) An abundance of conserved sites under purifying selection based on a substitution rate of 0.15 compared with the synonymous substitution rate of 0.21 in the *MLS1* coding region (Fay and Benavides 2005); 2) no significant alignment between *S. cerevisiae* and the more distantly related non-*Saccharomyces* species compared with alignment of scrambled sequences (Venkataram and Fay 2010); and 3) good matches to known binding sites in most of the species’ promoter sequence (fig. 1 and supplementary fig. S1, Supplementary Material online). Binding sites known to regulate *MLS1* expression in *S. cerevisiae* are two activation sites, which could be bound by either Cat8 or Sip4, a Mig1 repression site, and two Abf1 sites thought to be involved in basal expression (Caspary et al 1997; Roth et al. 2004). Although the number, position, and orientation of matching binding sites are different in all but the *Saccharomyces* species, they contain good matches to the known binding sites. The one exception is *Naumovozyma castellii*, which lacks a good TATA and Mig1 site. However, the binding site scores tend to be lower in more distantly related species, as measured by the total binding affinity predicted for each promoter (supplementary table S1, Supplementary Material online).

To examine potential differences in nucleosome occuppancy, we used a sequence-based prediction method (Kaplan et al. 2009), which matches in vivo measurements of nucleosome occuppancy at *MLS1* in *S. cerevisiae*. Similar to other noisy promoters (Blake et al. 2006), *MLS1* is characterized by a TATA element with nucleosomes positioned over its other binding sites in the presence of glucose. In ethanol, in vivo nucleosome occupancy goes down but not completely, typical of noisy gene expression found for many condition-specific genes (Kaplan et al. 2009). Occupancy predictions for the other yeast species are similar in that binding sites are often occupied (fig. 1). With the exception of Mig1 sites, the occupancy of binding sites in the non-*Saccharomyces* species are significantly higher than the median occupancy for each promoter (supplementary table S2, Supplementary Material online).

### Conserved Regulatory Patterns Despite Changes in Gene Expression Levels

**Fig. 2.**
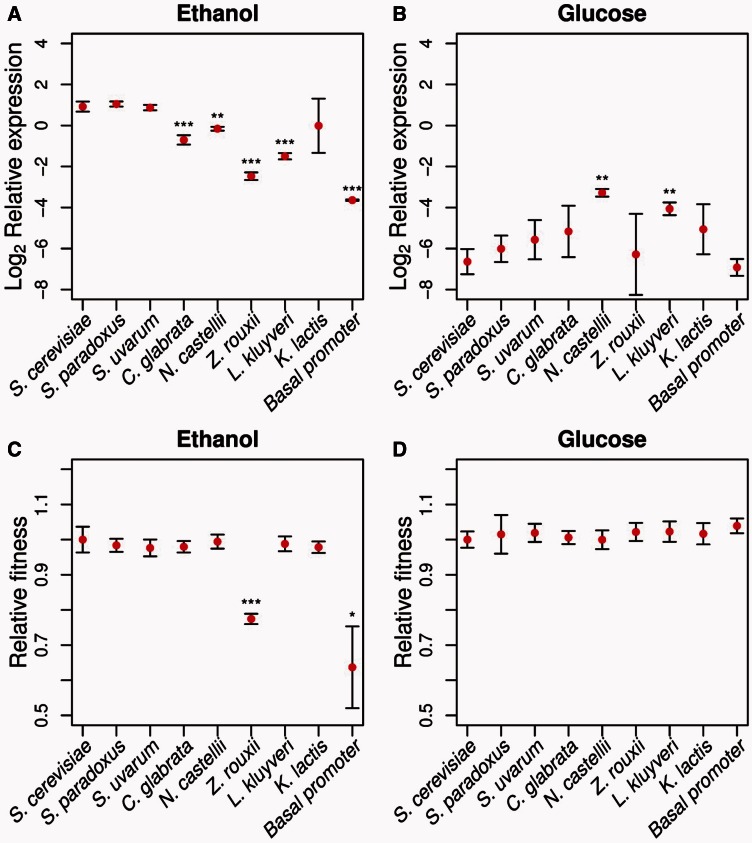
Gene expression and fitness of *MLS1* promoters in two environments. Relative expression shows expression of each species’ *MLS1* promoter and *Saccharomyces cerevisiae*’s basal promoter relative to the housekeeping gene *ACT1* on a log_2_ scale in (*A*) 3% ethanol and (*B*) 2% glucose. Relative fitness represents the growth rate of each strain relative to a reference competitor strain in (*C*) 3% ethanol and (*D*) 2% glucose. Error bars indicate one standard deviation, and significant differences in comparison with *S. cerevisiae* are shown for Bonferroni corrected *P < 0.05, **P < 0.01, and ***P < 0.001.

To test whether differences in the position, orientation, and slight changes in binding affinity affect gene expression, we placed each of the eight species’ noncoding regions upstream of the *S. cerevisiae MLS1* gene integrated at the *URA3* locus to avoid any confounding fitness effects caused by *DCP2* which is divergently transcribed from the same intergenic region as *MLS1*. All the promoters caused significant activation of *MLS1* in ethanol compared with glucose, ranging from 5.9- to 188-fold increase in expression (supplementary table S3, Supplementary Material online), demonstrating conservation of the response to carbon source. However, there is a general trend of a loss of both repression and activation in the most distantly related species (fig. 2A and *B*). Except for *K. lactis*, all non-*Saccharomyces* species’ promoters drove significantly lower expression compared with *S. cerevisiae* (Bonferroni correct *P* value < 0.05; fig. 2A). In glucose, both *N. castellii* and *Lachancea kluyveri* were not as well repressed as *S. cerevisiae* (Bonferroni corrected *P* value < 0.05; fig. 2B). Interestingly, the *N. castellii* promoter does not contain either a Mig1 repressor site (fig. 1) or a proximal TATA element (supplementary fig. S1, Supplementary Material online). The absence of a TATA box is known to correspond with a small dynamic range of expression (Basehoar et al. 2004).

To gauge the extent to which the activity of the *S. cerevisiae MLS1* promoter is influenced by known binding sites, we compared the *S. cerevisiae* promoter with the following: 1) A promoter lacking the *MLS1* proximal Cat8/Sip4 site, previously shown to have a larger effect than the distal site (Caspary et al. 1997), 2) a promoter lacking both Cat8/Sip4 sites, 3) a promoter lacking the Mig1 site, and 4) a basal promoter containing only the proximal 186 bp of the promoter, which includes TATA but lacks the Abf1, Cat8, and Mig1 sites (supplementary fig. S2, Supplementary Material online). Although deletion of either the proximal or both Cat8/Sip4 sites did not affect expression (supplementary fig. S3*A*, Supplementary Material online), the basal promoter drove expression at much lower levels in ethanol (fig. 2A). However, the basal promoter still caused a 9.7-fold increase in expression in ethanol compared with glucose, similar to the level of activation found for the promoters of *N. castellii*, *L. kluyveri*, and *Zygosaccharomyces rouxii* (fig. 2A and B and supplementary table S3, Supplementary Material online). Similar to a previous study (Caspary et al. 1997), the Mig1 deletion caused a loss of repression (supplementary fig. S3*B*, Supplementary Material online).

### Fitness Is Maintained Despite Changes in Gene Expression Levels

We tested whether any of the differences in expression affect fitness by competing each strain bearing a different *MLS1* promoter with a common reference strain in either glucose or ethanol. There were no significant differences in fitness between the *S. cerevisiae* promoter and that of any other species except for *Z. rouxii* in ethanol (fig. 2C and D). This indicates that with respect to gene expression levels in ethanol, there is a fitness plateau and a sharp cliff between the expression levels of *L. kluyveri* and *Z. rouxii* (fig. 2A and supplementary fig. S4, Supplementary Material online). The expression to fitness relationship for the *S. cerevisiae* promoter deletions are consistent with this fitness plateau (fig. 2 and supplementary fig. S3, Supplementary Material online). Deletion of all binding sites except the basal promoter had a large impact on fitness, consistent with its low expression level, and deletion of the Sip4/Cat8 and Mig1 sites had little to no impact on fitness in ethanol and a slight increase in fitness in glucose (supplementary fig. S3*C*, Supplementary Material online). Compared with an *S. cerevisiae* strain with *MLS1* at its endogenous locus, the transgenic *S. cerevisiae* allele of *MLS1* at the *URA3* locus exhibited a decrease in expression in ethanol, an increase in fitness in ethanol, and a decrease in fitness in glucose (supplementary fig. S3, Supplementary Material online). The fitness increase of the constructs integrated at the *URA3* locus was unexpected, but may be due to the lack of a functional *URA3* gene in the strain with *MLS1* at its endogenous position.

### Dynamic Expression and Fitness in Fluctuating Environments

**Fig. 3.**
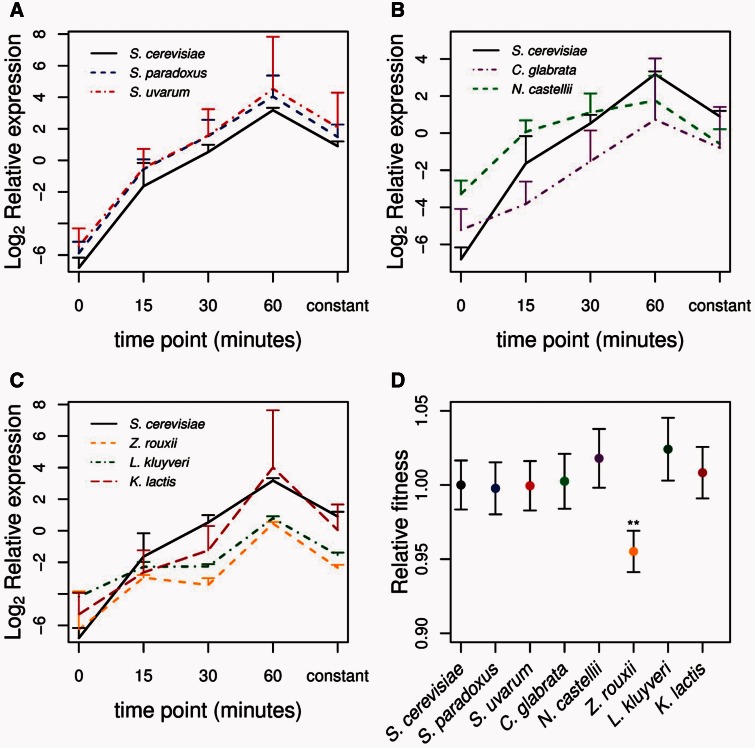
Gene expression and fitness of *MLS1* promoters in a fluctuating environment. Changes in *MLS1* expression for the eight species promoter constructs are shown in *A*–*C* and are divided into (*A*) *Saccharomyces* species, (*B*) post–whole-genome duplication species, and (*C*) pre–whole-genome duplication species. Relative expression levels represent the level of *MLS1* relative to the housekeeping gene *ACT1* on a log_2_ scale. Time point 0 is expression in complete media with 2% glucose and subsequent time points are expression levels 15, 30, and 60 min after being placed in complete media with 3% ethanol. The “constant” time point indicates expression after 24 h of exponential growth in ethanol media. Error bars indicate one standard deviation. Relative fitness of each *MLS1* species promoter construct (*D*) measured as the growth rate of each strain relative to a fluorescent competitor strain after 3 days of sequential competition in complete media with 3% ethanol plus 0.2% glucose. Bonferroni corrected *P* values are indicated for *P < 0.05, **P < 0.01, and ***P < 0.001.

Our previous measurements of gene expression levels and fitness were done during exponential growth after cells were allowed to condition themselves to growth on glucose or ethanol. However, the dynamic response of a promoter to different carbon sources may be as important to fitness as expression levels after adjustment to a single condition. To examine the temporal dynamics of each species’ promoter, we measured expression following a switch from growth on glucose to ethanol. Similar to expression levels after acclimation, expression dynamics after switching from glucose to ethanol are conserved within the *Saccharomyces* species as is apparent from the consistent response over time (fig. 3A). For all the non-*Saccharomyces* species, we observed a smaller increase in expression between 0 and 15 min after switching to growth on ethanol (fig. 3B and C and supplementary table S6, Supplementary Material online). *Zygosaccharomyces* *rouxii* and *L. kluyveri* also showed a smaller increase in expression between 15 and 30 min (fig. 3B and C and supplementary table S6, Supplementary Material online). Although the dampened response of non-*Saccharomyces* species to ethanol is consistent with their lower expression levels after acclimation to ethanol media (fig. 1A), the absolute expression level at 15 min was only different between *S. cerevisiae* and *Candida glabrata* (supplementary table S7, Supplementary Material online).

Given the different expression dynamics, we tested whether the *MLS1* promoters cause fitness differences in an environment where cells must switch to growth on ethanol once all the glucose has been used. Only the *Z. rouxi* promoter showed significantly reduced fitness (Bonferroni corrected *P* value < 0.05; fig. 3D), the only species with lower fitness in a constant ethanol environment (fig. 1C). The higher fitness of the *Z. rouxii* promoter in a fluctuating carbon source environment, compared with a constant environment, is likely a result of the competition including growth in glucose where no fitness defect was measured. In the fluctuating carbon source environment, there was a slightly higher fitness for the promoter of *L. kluyveri* (fig. 3D), potentially caused by the weaker *MLS1* repression in glucose enabling the strain with the *L. kluyveri* promoter to start growing earlier (fig. 1B). The other species’ promoter with a significant loss of repression in glucose, *N. castellii* (fig. 1B), had higher fitness in the fluctuating carbon source environment that is close to significant (fig. 3D).

## Discussion

Knowing how gene expression affects fitness is important to interpreting patterns of gene expression divergence. Using *MLS1* promoters from eight yeast species, we find that large differences in gene expression levels do not generate detectable fitness effects. However, we also find a large drop in fitness below a certain low level of expression, implying that the *S. cerevisiae MLS1* promoter resides on a fitness plateau. The high fitness of various configurations of binding sites present in different species provides further experimental support for the flexibility of the *cis*-regulatory code.

### Conservation of Carbon Source Response Combined with Divergence in Expression Levels

Similar to previous promoter studies (Gordon and Ruvinsky 2012), we find that *MLS1* promoters are conserved in their ability to respond to glucose and ethanol but also exhibit loss of both activation and repression as divergence between the promoters of these species and *S. cerevisiae* increases. Also consistent with a previous study of interspecific divergence (Tirosh et al. 2008), we find little correspondence between changes in binding sites and expression levels. First, deletion of one or both Cat8/Sip4 sites did not affect expression and there is no strong correlation between the summed binding affinity of a promoter and its expression level (supplementary table S1, Supplementary Material online). A previous study (Caspary et al. 1997) found that mutation of the proximal Cat8/Sip4 site or both Cat8/Sip4 sites caused a 28% and 80% reduction in expression, respectively. One explanation for why we did not find effects for these sites is that our deletion constructs altered the spacing of other binding sites. For example, Abf1 sites were brought close to TATA. However, the different results could also be a consequence of Caspary et al. (1997) measuring expression from a high-copy episomal plasmid using a reporter assay rather than *MLS1* itself, whereas we measured expression of *MLS1* integrated into the *URA3* locus. A second line of evidence for the importance of sequences besides Cat8/Sip4 sites is that the basal promoter still yielded a 9.7-fold increase in expression in ethanol compared with glucose (supplementary table S3, Supplementary Material online). However, we did find effects associated with the Mig1 binding site: The Mig1 deletion caused a loss of repression in the *S. cerevisiae* promoter and *N. castellii* had the highest expression in glucose and also lacked a Mig1 site.

### The Fitness-Expression Function in *Saccharomyces* *cerevisiae*

Previous work has shown condition-specific fitness costs and benefits of Lac expression in bacteria (Dekel and Alon 2005; Perfeito et al. 2011), and a fitness plateau for *LCB2* expression in yeast (Rest et al. 2013). Results for *MLS1* expression differ from *LCB2* in that endogenous *LCB2* expression levels occurred at the edge of the fitness cliff whereas no detectable loss of fitness occurred for up to a 5.4-fold (*L. kluyveri*) drop below wild-type levels for *MLS1*. We put forth four explanations for the high level of *MLS1* expression in *S. cerevisiae*. First, low *MLS1* expression levels may cause reduced fitness in conditions other than those measured. For example, *MLS1* is required for sporulation and low expression could reduce or alter sporulation efficiency. Second, high *MLS1* expression could be maintained by small fitness effects that are not detectable by our assays. Because purifying selection can occur on selection coefficients as small as the inverse of the effective population size, the fitness plateau could be covered with undetectable hills. In support of this possibility, there is good evidence for purifying selection on *MLS1* binding sites within the *Saccharomyces* species (supplementary fig. S5, Supplementary Material online; see also Doniger and Fay 2007). Third, the fitness-expression function may only have a small plateau in other genetic backgrounds. Strain differences in *LCB2* expression imply that genetic background modulates the fitness-expression function (Rest et al. 2013). Finally, high *MLS1* expression may be due to genetic constraints whereby mutations which could lower the expression of *MLS1* are constrained by pleiotropic effects on other genes. For instance, mutations may also lower the expression of coregulated genes.

What types of expression changes affect fitness? In addition to expression levels, prior work in yeast showed that noise in *TDH3* expression affects fitness (Metzger et al. 2015). *MLS1* like other TATA-containing promoters is characterized by large variation in cell-to-cell levels of expression, which may provide a fitness advantage under fluctuating environments through bet hedging (Thattai and Van Oudenaarden 2004; Kaern et al. 2005; Solopova et al. 2014). However, we found fitness effects in a fluctuating carbon source environment (fig. 3) to mirror and be smaller than those under exponential growth on a single carbon source (fig. 2).

One limitation of our approach is that we used heterologous expression and fitness assays. As such, it is possible that *MLS1* promoters from the distantly related yeast species do not have reduced activation and repression in their endogenous genome. Both the extensive *cis*–*trans* expression interactions found to occur between species (McManus et al. 2010; Swain Lenz et al. 2014) and the dependency of the fitness-expression function on strain background (Rest et al. 2013) indicate that endogenous *MLS1* expression in other species may not be the same as that measured in *S. cerevisiae*. However, a prior study of gene expression following the diauxic shift found that, with the exception of *N. castellii*, *MLS1* is consistently actived between 3.7- and 8.6-fold (Thompson et al. 2013; supplementary table S4, Supplementary Material online). With the exception of *SIP4* in *Saccharomyces* *uvarum*, *CAT8* and *SIP4* are also induced following the diauxic shift. Interestingly, comparing the *MLS1* fold change from glucose to ethanol from figure 2 to the fold change during the diauxic shift in supplementary table S4, Supplementary Material online, shows a strong correlation between heterologuous and endogenous *MLS1* species’ promoters (supplementary table S5, Supplementary Material online). These data support the possibility that the expression divergence observed in figure 2 is representative of the *MLS1* expression divergence that has occurred between these species.

Another limitation of the heterologous assays is that we do not know whether the expression fitness function has changed between species. Different species could have different optimal levels of *MLS1* expression. For example, the optimal expression of *MLS1* in *Z. rouxii* could be quite low. However, without measurements of endogenous expression in *Z. rouxii*, it is hard to know whether this is the case. Thus, our use of heterologous measurements limits our interpretations to how different promoters with different outputs affect fitness.

In conclusion, our finding of a fitness plateau for *MLS1* expression provides an explanation for divergence in gene expression levels and configurations of binding sites without an overall change in carbon source response. Current models for the evolution of *cis*-regulatory sequences hypothesize neutral evolution with a constant transcriptional output. However, when fitness effects are small or absent, many changes in *cis*-regulatory sequences may evolve under a neutral model despite their effects on gene expression.

## Materials and Methods

### Binding Site and Nucleosome Predictions

Position weight matrices (PWMs) for Abf1, Sip4, and Mig1 were obtained from MacIsaac et al. (2006). The PWM for Cat8 was obtained from a curated list of motifs (Soontorngun et al. 2007), and the PWM for TATA (NHP6A) was from Zhu et al. (2009). Sequences were searched for binding sites using Patser (Hertz and Stormo 1999). Only binding site scores below a ln(*P* value) of 7 were considered, where the *P* value is the expected probability of a random match to the binding site (Hertz and Stormo 1999). Nucleosome occupancy probability was predicted for each *MLS1* promoter (Kaplan et al. 2009). The temperature and histone concentration parameters were set to 1 and 0.03, respectively, as in Kaplan et al. (2009).

A single sample Wilcoxon signed-rank test was used to determine if histone occupancy probabilities were higher at binding site positions than the median occupancy of the promoter (supplementary table S2, Supplementary Material online). First, the median occupancy for each promoter was subtracted from the occupancy probability at each binding site. Next, binding sites from the non-*Saccharomyces* species were pooled and tested for the alternative hypothesis that average occupancy at these sites was greater than the median occupancy. Only non-*Saccharomyces* species were used because these sequences are more or less phylogenetically independent based on their lack of sequence homology. Each transcription factor and TATA were tested separately.

### Species Promoter Constructs

*MLS1* promoter regions from eight species were placed into an *S. cerevisiae* background. First, the pRS306-ScMLS1 plasmid was constructed by inserting *MLS1* from *S. cerevisiae* (S288c) into the integrative plasmid pRS306 (Sikorski and Hieter 1989). The *MLS1* region from S288c includes the 893-bp noncoding region upstream of *MLS1* as well as the 305-bp region downstream of the *MLS1* translation stop site. Second, the promoter of *S. cerevisiae MLS1* in pRS306-ScMLS1 was then replaced by the *MLS1* promoter in seven other yeast species in the following manner. *MLS1* promoter regions were defined as the noncoding region upstream of the *MLS1* start codon to the beginning of the next coding region. In the cases of *N. castellii* and *Z. rouxii*, the predicted intergenic regions were short (575 bp for *Z. rouxii* and 250 bp for *N. castellii*) and therefore the region used for these two promoters was ∼1 kb upstream of the *MLS1* start codon. The promoter region of *MLS1* from each species (supplementary table S8, Supplementary Material online) was PCR amplified (see supplementary file S1, Supplementary Material online, for primers) and subcloned into the pRS306-ScMLS1 plasmid using the Gibson Assembly method (New England Biolabs, Ipswich, MA). The promoter region as well as the *S. cerevisiae MLS1* coding region were sequence confirmed for each construct.

### Binding Site Deletions

Binding sites were deleted by removing the region surrounding each binding site from the promoter. Regions deleted are shown in supplementary figure S2, Supplementary Material online. Deletions were generated by amplifying the pRS306-ScMLS1 plasmid with segments of the promoter missing. Primers contained BglII sites on their 5′ end (supplementary file S1, Supplementary Material online). After amplification, the PCR product was digested with BglII and ligated back together to form a circular plasmid. The *S. cerevisiae MLS1* promoter deletions and coding region were sequence confirmed.

### Plasmid Integrations

The endogenous *MLS1* coding region from the strain YJF186 (YPS163 oak isolate, Mat *a*, HO::dsdAMX4, ura3-140) was deleted by replacement with the KANMX4 cassette to generate the strain YJF604. All pRS306-based plasmids described above were cut in the URA3 coding region with StuI and integrated into YJF604 using lithium acetate transformation (Geitz and Woods 2002) and selected on plates lacking uracil. The competitor strain containing yellow fluorescent protein (YFP) was generated by integrating a YFP-NATMX4 plasmid-containing homology to the HO locus (received from R. Kishony) into YJF186.

### Competitive Fitness Assays

Fitness was estimated by competing each strain against a YFP marked reference strain. For each competition, six biological replicates (independent transformants) of each integrated construct were competed against the YFP competitor at 30 °C at 300 rpm in 3 ml media in 18 × 150 mm glass tubes. Ethanol (3%), glucose (2%), and mixed carbon source (3% ethanol and 0.2% glucose) competitions were carried out in complete medium (CM: 0.67% (wt/vol) nitrogen base with ammonium sulfate and amino acids) with the specified carbon sources. All strains were acclimated to each growth medium prior to competition by 3 days of growth, with cells resuspended in fresh medium after each day at an OD_600_ of 0.07. An OD_600_ of 1 is ∼10^7^ cells/ml. The YFP competitor strain was mixed with each culture at a 50:50 ratio at a starting cell density of 0.7 × 10^6^ cells/ml. All measurements were taken within the linear range of an OD_600_ between 1 and 0.1. Competitions in CM with 3% ethanol and CM with 3% ethanol + 0.2% glucose were carried out for 2 days with resuspension in fresh medium after every 23 h of competition. Competitions in CM with 2% glucose were carried out for 1 day with resuspension in fresh medium after every 11 h of competition. There were approximately the same number of generations for the competitions grown in all media (between 8.5 and 9.5 generations). All cultures were switched to new media during exponential growth and never allowed to reach saturation. The proportion of YFP positive strains was determine at the beginning and end of each competition. Cells from each culture were also diluted to an OD_600_ = 0.2 in sheath fluid and run on a Beckman Coulter FC 500 MPL flow cytometer (Beckman Coulter, Brea, CA). For each sample, 20,000 cells were counted and gated to distinguish between fluorescent and nonfluorescent cells. The false negative rate for YFP cells was 0.003.

### Fitness Calculations

Fitness measurements were calculated using $w_{i}=ln\left(N_{1}/Y_{1}\right)-ln\left(N_{0}/Y_{o}\right)$ as in Hartl and Clark (1997), where $Y_{o}$ and $N_{o}$ are the starting frequencies of the YFP strain and the nonfluorescent competitor strain, respectively. Here,$\;N_{o}=1-Y_{o}$. Similarly, $N_{1}$ and $Y_{1}$ represent these frequencies at the end of the competition. Relative fitness of a given strain i is equal to $w_{i}/{\bar{w}}_{scer}$, where ${\bar{w}}_{scer}$ is the average fitness of the *S. cerevisiae* strains.

### *MLS1* mRNA Expression Analysis

*MLS1* measurements during exponential growth were measured as follows. Using four of the same replicates for each promoter from the competition, each strain was acclimated and cells were sampled 4 h after being resuspended in fresh CM with 3% ethanol or CM with 2% glucose medium at an OD_600_ of 1 on the third day. The equivalent of 1 ml cells at an OD_600_ of 0.3 were sampled. Cells were centrifuged, supernatant was removed, pellets were frozen in liquid nitrogen, and stored at −80 °C.

*MLS1* mRNA expression during the switch from glucose to ethanol was obtained from 4 time points. After 3 days acclimation cells were placed in 3 ml CM with 2% glucose at an OD_600_ = 1 and grown for 4 h. Cells were centrifuged for 30 s at 3,000 rpm, supernatant was removed and cells were washed with 1 ml CM with 3% ethanol and centrifuged again. Supernatant was removed and cells were resuspended in 3 ml of CM with 3% ethanol and cultures were placed in the incubator. Cells were then sampled 15, 30, and 60 min after cells were initially placed into CM with 3% ethanol, centrifuged, and pellets were frozen in liquid nitrogen and stored at −80 °C.

*MLS1* expression was measured using QuantiGene (Affymetrix, Santa Clara, CA) following manufacturer’s instructions. In total, 200 μl of homogenization buffer (Affymetrix) was added to each pellet, resuspended, centrigued, and supernatant was removed. Pellels were resuspended in 100 μl of ZYM buffer (Clontech, Mountain View, CA) and 10 μl of zymolase (Clontech) and allowed to digest for 1 h at 30 °C at 300 rpm. After digestion, 150 μl of homogeniztion buffer was added to each well. The content of each well was then diluted 1:100 in homogenization buffer. Next, 40 μl of these 1:100 diluted samples were added to 60 μl of “working bead mix” described in Steps 4–6 of the “Purified RNA or in vitro Transcribed RNA” protocol in the QuantiGene 2.0 Plex Assay User Manual (Panomics Solutions P/N 16659 Rev.C 020912). The Purified RNA or in vitro Transcribed RNA protocol was then followed exactly from Step 7 onwards. Probes were designed to the *MLS1* and *ACT1* coding regions of *S. cerevisiae*. A total of 40 μl of the 1:100 diluted samples was added to 60 μl of mastermix. Measurements were obtained on a Bio-Plex 200 System (Life technologies, Carlsbad, CA) and analyzed using the Bio-Plex Manager 6.1 software. Standard curves for each analyte were generated by a 4-fold serial dilution of one of the *S. cerevisiae MLS1* promoter strains sampled in ethanol media.

### Statistical Analysis of Fitness and Expression

Six biological replicates (independent integrations at the URA3 locus) of each promoter construct were measured for fitness. Four biological replicates of each promoter were measured for expression in exponential growth after acclimation to either glucose or ethanol. Outliers from each group were removed using the Grubbs’ test (*P* < 0.05). Significant differences were measured by *t*-tests with unequal variance. Bonferonni correction was used for the seven hypotheses that another species promoter was different than the *S. cerevisiae* promoter. For measurements of the dynamics of gene expression from glucose to ethanol, three biological replicates were used and no outliers were removed. A nested analysis of variance was used to measure the differences between each species’ promoter at each time point as well as the rate of change (slope) between each time point. This was done in R where level∼(species/time).

## Supplementary Material

Supplementary tables S1–S8, figures S1–S5, and file S1 are available at Molecular Biology and Evolution online (http://www.mbe.oxfordjournals.org/).

Supplementary Data
