# Supplementary material for: Divergent MLS1 Promoters Lie on a Fitness Plateau for Gene Expression
Source: Mol Biol Evol. 2016 Jan 18;33(5):1270–9. doi: 10.1093/molbev/msw010 (PMC4839218; doi:10.1093/molbev/msw010)
Supplement: Supplementary Data [file supp_33_5_1270__index.html]

Divergent MLS1 Promoters Lie on a Fitness Plateau for Gene Expression — Divergent MLS1 Promoters Lie on a Fitness Plateau for Gene Expression — Supplementary Data 

# Divergent *MLS1* Promoters Lie on a Fitness Plateau for Gene Expression

## Supplementary Data

files

- Supplementary Data - pdf file
