# Supplementary material for: Divergent MLS1 Promoters Lie on a Fitness Plateau for Gene Expression
Source: Mol Biol Evol. 2016 Jan 18;33(5):1270–9. doi: 10.1093/molbev/msw010 (PMC4839218; doi:10.1093/molbev/msw010)
Supplement: Supplementary Data [file supp_msw010_Supporting_Info_Dec9_Revision.pdf]

Supporting Information

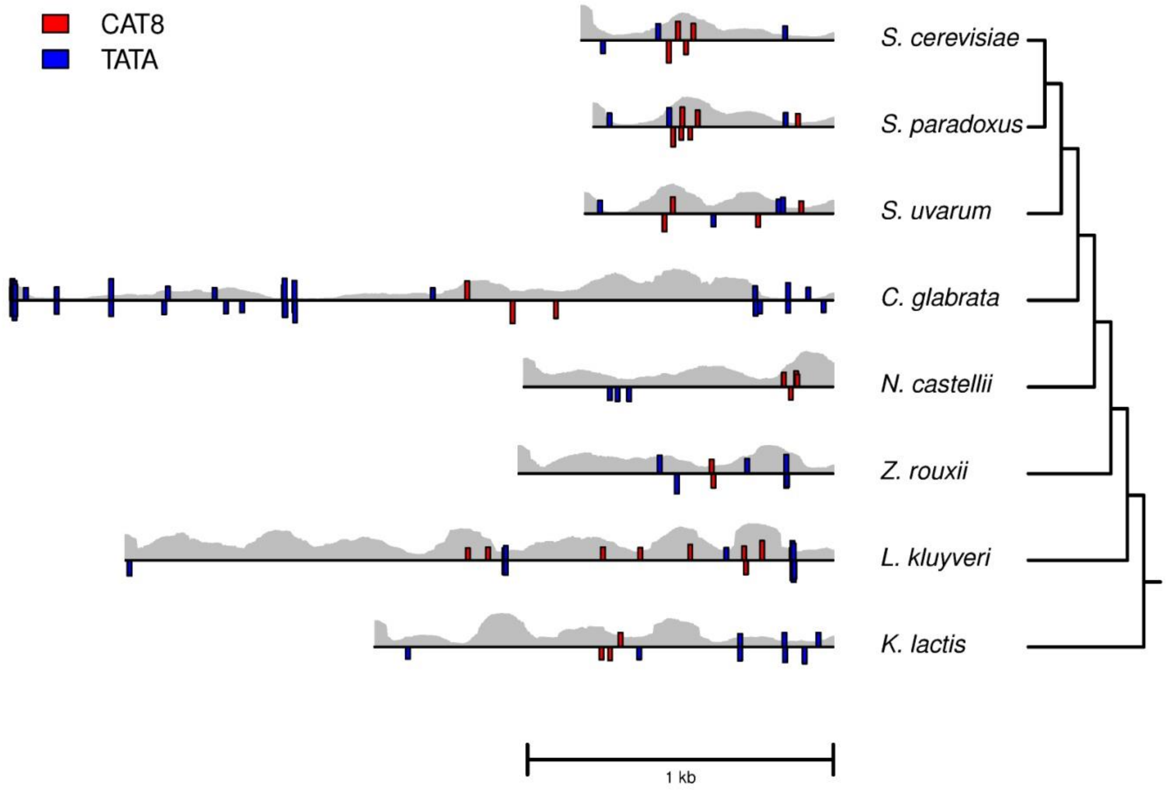

Figure S1. Transcription factor binding and nucleosome occupancy predictions for *MLS1* promoters. The noncoding region upstream of *MLS1* from eight yeast species, where the heights of colored bars represent the scores of predicted binding sites for Cat8 and TATA. Bars above each line represent sites on the forward strand and those below represent sites on the reverse strand. The probability of nucleosome occupancy at each base pair along the promoters is represented by the height of the grey bars in the background.

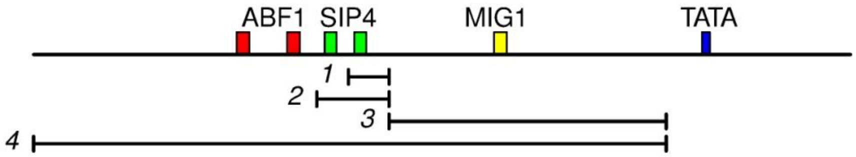

Figure S2. Regions deleted from the *S. cerevisiae MLS1* promoter. Each bar indicates a deleted region. The deletions are 1) one Sip4/Cat8 binding site 2) two Sip4/Cat8 binding sites 3) the region surrounding the Mig1 binding site and 4) the entire promoter except the basal promoter.

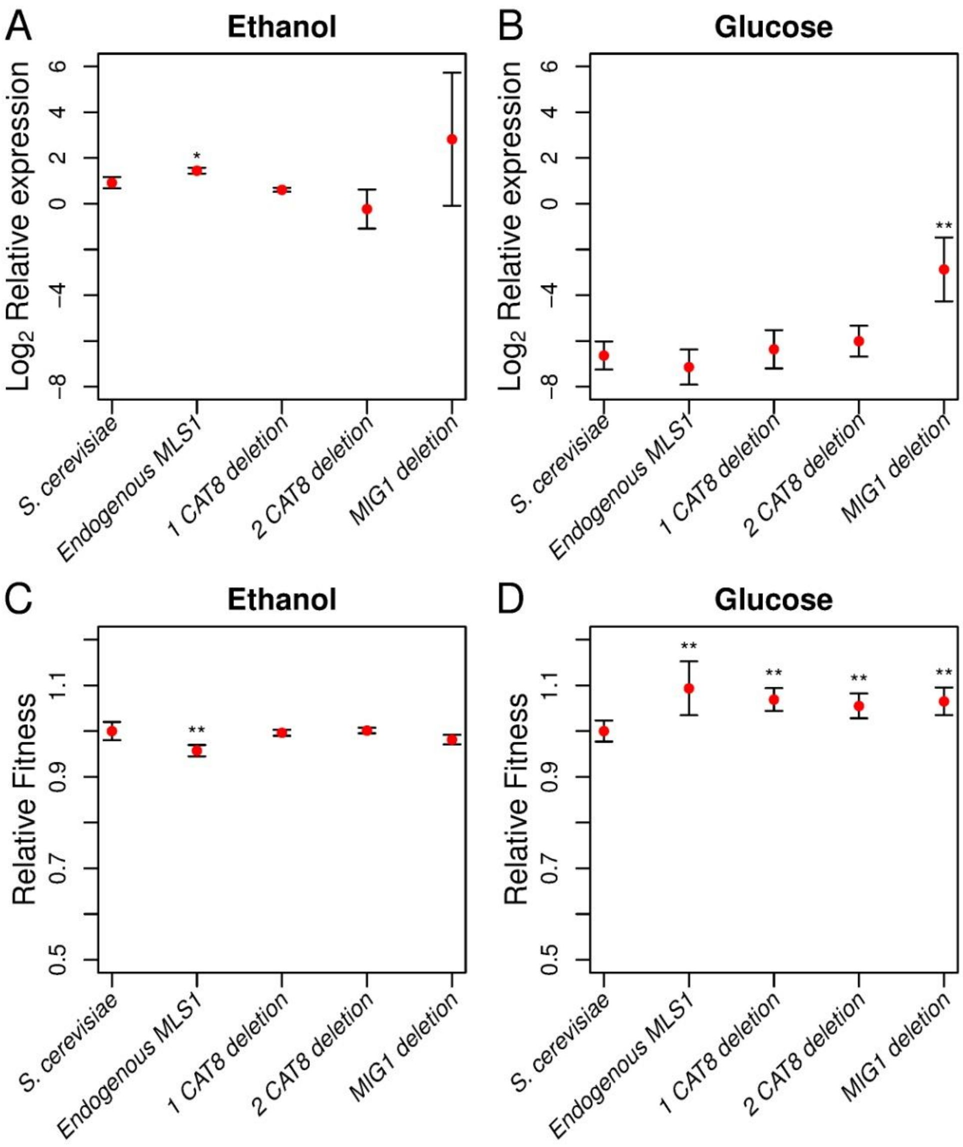

Figure S3. Gene expression and fitness of *MLS1* deletion promoters in two environments. Relative expression shows expression of each *MLS1* promoter construct relative to the housekeeping gene *ACT1* on a $\log_2$ scale in (A) 3% ethanol and (B) 2% glucose. Relative fitness represents the growth rate of each strain relative to a reference competitor strain in (C) 3% ethanol and (D) 2% glucose. Error bars indicate one standard deviation, significant differences in comparison to the wildtype *S. cerevisiae* promoter (*S. cerevisiae*) are shown for P value <0.05 (*), P<0.01 (**) and P<0.001 (***). For reference, *MLS1* expression and fitness are also shown from its endogenous locus (Endogenous *MLS1*).

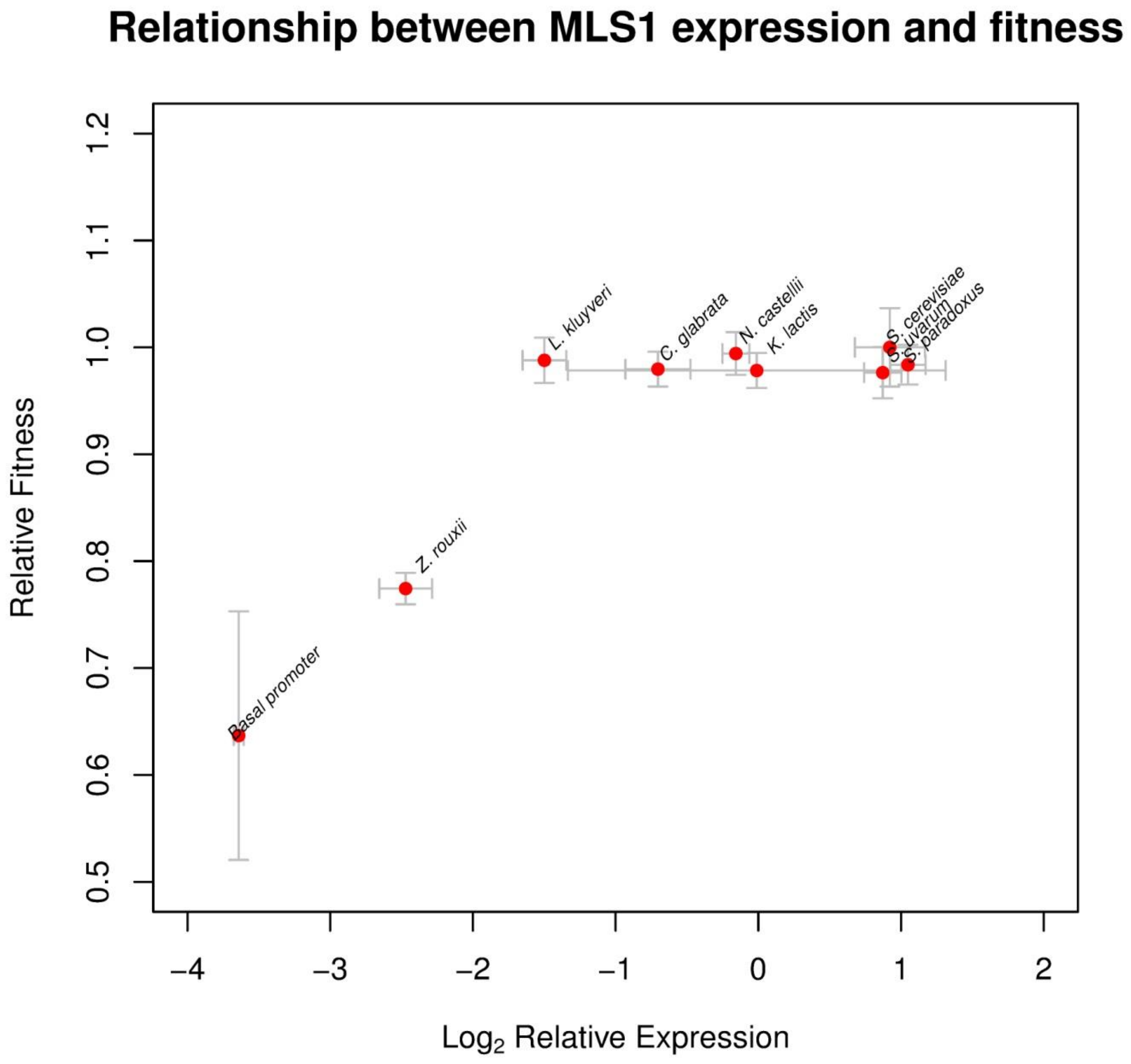

Figure S4. Relationship between *MLS1* gene expression and fitness in ethanol. The fitness data from Figure 2C is plotted against the *MLS1* expression data from Figure 2A. As in Figure 2, relative expression represents expression of each *MLS1* promoter construct relative to the housekeeping gene *ACT1* on a $\log_2$ scale. Relative fitness represents the growth rate of each strain relative to a reference competitor. Error bars indicate one standard deviation.

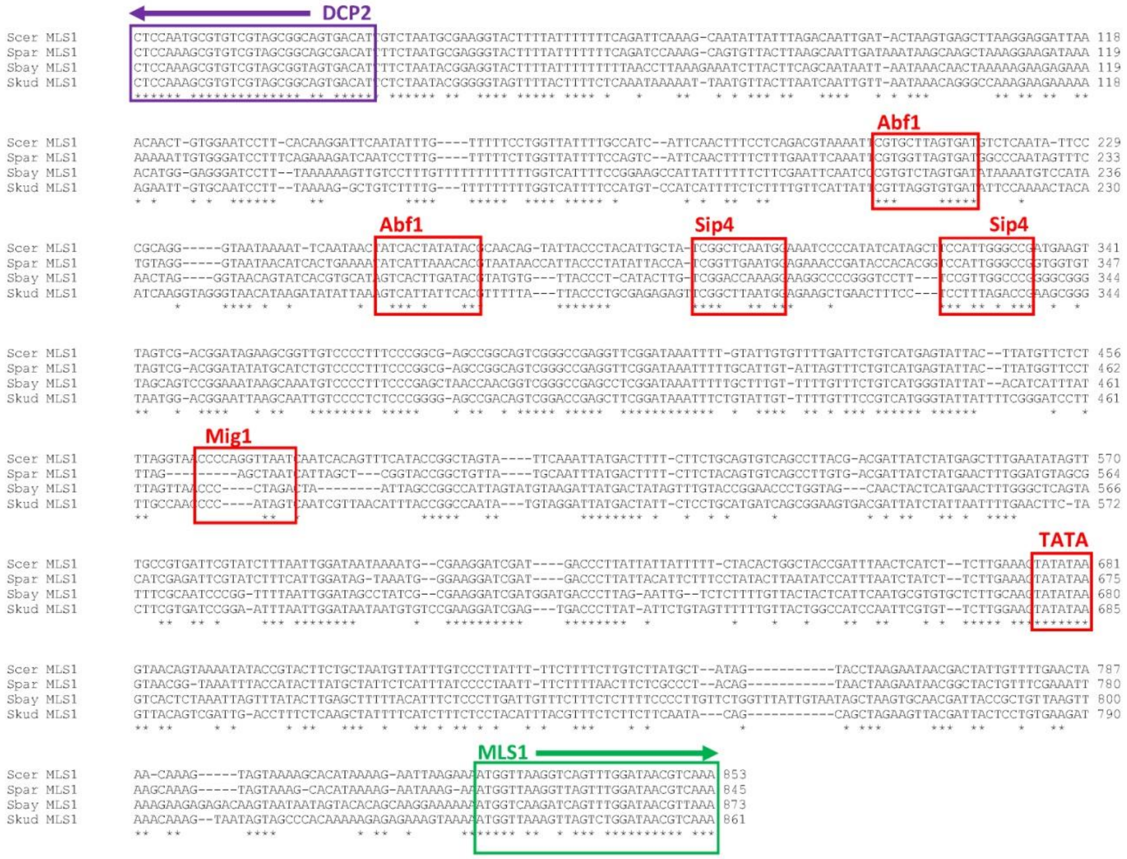

Figure S5. Alignment of *MLS1* promoter regions among *Saccharomyces sensu stricto* species. A ClustalW alignment is shown for the *MLS1* promoters for *S. cerivisiae, S. paradoxus, S. uvarum* and *S. mikatae.* Known binding sites are boxed in red, *MLS1* and *DCP2* coding regions are boxed in green and purple, respectively. Stars indicate sites that are conserved between all four species.

Table S1. The sum of all position weight matrix scores for each *MLS1* promoter.

	ABF1	SIP4	MIG1
S. cerevisiae	23.7	24.7	11.6
S. paradoxus	21.3	34.9	5.3
S. uvarum	25.6	19.5	10.6
C. glabrata	29.2	24.2	0
N. castellii	7.1	8.1	19.0
Z. rouxii	21.5	7.9	11.6
L. kluveri	33.8	18.8	16.9
K. lactis	9.3	8.9	5.3

Scores represent the sum of all PWM scores above a cutoff of ln(p-value)=-7. This cutoff corresponds to scores of 2.427, 0.808, and 5.222 for ABF1, SIP4, and MIG1, respectively.

Table S2. Single sample Wilcoxon signed-rank test for histone occupancy at binding sites relative to the promoter median histone occupancy.

Transcription factor [a]	Alternative hypothesis for binding site occupancy [b]	p-value
Sip4	greater than promoter median	0.0087
Abf1	greater than promoter median	0.016
Mig1	greater than promoter median	0.25
Cat8	greater than promoter median	0.0014
TATA	less than promoter median	0.0013

[a] Each transcription factor represents all sites in the five non-*Saccharomyces* species studied.

[b] See the Materials and Methods section for details on the single sample Wilcoxon signed-rank test.

Table S3. Absolute difference of log2 relative expression between ethanol and glucose media for each strain.

	Log_2 relative expression[a]			Glucose to Ethanol fold change (non-log_2 scale)
	Ethanol	Glucose	Difference	
S. cerevisiae	0.92	-6.64	7.56	188.7
S. paradoxus	1.05	-6.01	7.06	133.4
S. uvarum	0.87	-5.57	6.44	86.8
C. glabrata	-0.70	-5.17	4.46	22.0
N. castellii	-0.16	-3.28	3.44	10.8
Z. rouxii	-2.47	-6.28	3.81	14.0
L. kluyveri	-1.50	-4.06	2.56	5.9
K. lactis	-0.01	-5.06	5.05	33.1
basal promoter	-3.64	-6.92	3.28	9.7
1 Cat8 deleted	0.61	-6.36	6.97	125.4
2 Cat8 deleted	-0.23	-6.01	5.78	54.9
Mig1 deleted	2.82	-2.87	5.69	51.6
Endogenous MLS1	1.45	-7.14	8.59	385.3

[a] Relative expression in ethanol and glucose correspond to the mean values shown in Figure 2 and Figure S3.

Table S4. Relative log_2 gene expression data from Thompson *et al.* (2013) during the switch from fermentative growth on glucose to aerobic respiration following the diauxic shift for *MLS1*, as well as the transcription factors *CAT8, SIP4, MIG1, ABF1* and the gluconeogenesis genes *MDH2* and *PCK1*.

Gene		Lag phase	Late Log	Diauxic Shift	Post Shift	Plateau	Fold change (Plateau -Lag)
MLS1	Scer	2.196	1.128	1.641	5.649	10.106	7.910
	Spar	2.760	0.180	1.271	9.617	11.018	8.259
	Smik	0.295	0.276	0.842	4.930	8.848	8.553
	Sbay	-0.491	0.559	2.584	3.092	3.188	3.678
	Sbay.uv	1.219	0.419	1.890	0.517	6.916	5.697
	Cgla	0.053	0.876	2.440	5.288	4.670	4.617
	Ncas	0.750	-0.177	-0.103	-0.172	0.915	0.165
	Kpol	3.533	5.858	8.147	9.578	11.270	7.737
	Kwal	1.968	-0.145	0.863	2.652	6.448	4.480
	Lklu	1.955	1.100	1.931	5.161	5.654	3.699
	Klac	0.458	0.202	2.552	3.756	5.986	5.527
	Calb	-2.028	2.589	4.641	5.321	6.212	8.240
	Ylip	-4.114	-1.290	2.486	2.775	3.310	7.424
CAT8	Scer	1.141	1.201	3.790	6.561	5.787	4.646
	Spar	-0.172	1.760	5.260	5.497	5.349	5.520
	Smik	-1.323	4.192	4.792	5.621	5.711	7.035
	Sbay	3.330	1.796	5.061	5.483	5.583	2.253
	Sbay.uv	-0.158	1.167	2.825	1.938	3.735	3.892
	Cgla	-0.530	1.025	2.074	5.048	3.397	3.927
	Ncas	3.241	-0.117	4.103	4.840	5.692	2.451
	Kpol	NA	NA	NA	NA	NA	
	Kwal	-0.153	0.296	1.025	1.512	2.051	2.204
	Lklu	-0.238	0.207	1.975	2.036	1.771	2.009
	Klac	1.123	0.413	0.683	0.823	0.952	-0.171
	Calb	0.811	3.385	1.681	2.119	1.940	1.129
	Ylip	NA	NA	NA	NA	NA	
SIP4	Scer	2.564	-0.177	0.531	6.935	8.104	5.541
	Spar	1.195	0.059	1.138	7.975	7.809	6.615
	Smik	2.997	2.365	3.603	6.179	7.148	4.151
	Sbay	-0.624	-1.319	-0.144	0.189	0.064	0.687
	Sbay.uv	2.211	-0.124	0.202	-0.124	2.673	0.462
	Cgla	1.271	-0.155	0.174	4.832	3.195	1.925
	Ncas	0.861	1.323	1.007	0.164	4.162	3.301
	Kpol	1.331	1.239	5.427	8.545	7.554	6.223

	Kwal	3.087	0.310	3.396	7.702	8.797	5.710
	Lklu	3.324	-0.060	5.113	7.813	7.148	3.824
	Klac	2.369	-0.435	2.318	4.779	5.919	3.551
	Calb	NA	NA	NA	NA	NA	
	Ylip	NA	NA	NA	NA	NA	
MIG1	Scer	1.001	-0.337	-0.254	-1.596	-1.906	-2.906
	Spar	0.664	0.076	-1.122	-2.624	-2.659	-3.323
	Smik	0.818	-0.729	-0.682	-1.366	-2.933	-3.751
	Sbay	0.905	0.157	-0.350	-0.495	-0.650	-1.555
	Sbay.uv	1.066	-0.377	-0.615	-0.608	-1.948	-3.014
	Cgla	1.985	0.236	-0.045	-1.897	-1.131	-3.116
	Ncas	-0.122	0.067	-0.379	-0.022	0.891	1.013
	Kpol	NA	NA	NA	NA	NA	
	Kwal	0.466	0.515	1.518	1.533	1.849	1.383
	Lklu	1.181	-0.025	0.686	0.951	1.648	0.467
	Klac	-1.676	-0.587	-0.328	-0.660	-0.060	1.616
	Calb	-0.537	0.103	1.228	1.523	1.421	1.958
	Ylip	0.938	0.086	2.681	2.301	2.118	1.180
ABF1	Scer	0.914	-0.441	-0.512	-2.791	-1.765	-2.678
	Spar	0.717	-0.126	-1.419	-1.268	-0.992	-1.708
	Smik	1.025	-0.762	-0.852	-1.175	-0.838	-1.863
	Sbay	0.119	-0.291	-0.824	-1.258	-1.629	-1.747
	Sbay.uv	-0.236	-0.262	-0.335	-0.251	-1.021	-0.785
	Cgla	NA	NA	NA	NA	NA	
	Ncas	-0.141	0.084	-0.510	-1.243	-1.248	-1.107
	Kpol	-0.056	-0.577	-0.184	-0.818	-0.563	-0.507
	Kwal	0.255	0.918	1.156	1.967	0.457	0.202
	Lklu	0.158	-0.073	-0.310	-0.088	-0.621	-0.779
	Klac	1.365	-0.366	-1.285	-1.431	-0.994	-2.359
	Calb	NA	NA	NA	NA	NA	
	Ylip	NA	NA	NA	NA	NA	
MDH2	Scer	0.774	1.683	2.256	5.255	7.261	6.488
	Spar	2.034	1.509	3.555	6.713	7.374	5.340
	Smik	1.482	0.614	1.318	2.363	5.592	4.110
	Sbay	1.062	0.339	2.055	2.201	1.903	0.841
	Sbay.uv	0.936	0.202	1.524	0.625	2.487	1.550
	Cgla	2.562	0.734	2.187	4.212	3.302	0.740
	Ncas	1.710	-0.116	0.628	1.330	2.964	1.254
	Kpol	0.830	0.231	1.218	2.483	3.639	2.809

	Kwal	1.850	0.651	4.065	5.986	5.786	3.936
	Lklu	-1.659	-0.697	0.887	1.692	0.653	2.312
	Klac	-3.018	-1.634	-1.722	-1.896	-3.224	-0.206
	Calb	-5.761	0.847	2.206	3.760	3.808	9.568
	Ylip	-3.253	0.382	-0.387	0.727	1.218	4.471
PCK1	Scer	4.994	0.493	0.871	7.704	12.214	7.220
	Spar	3.733	0.190	0.358	10.540	10.716	6.983
	Smik	1.221	0.487	2.024	7.149	12.139	10.918
	Sbay	1.802	0.084	1.325	2.207	3.150	1.348
	Sbay.uv	5.536	-0.757	1.300	-0.381	8.178	2.643
	Cgla	5.140	0.550	2.243	6.621	8.749	3.608
	Ncas	0.487	1.425	1.501	1.825	4.292	3.805
	Kpol	3.256	3.341	7.310	9.728	11.625	8.369
	Kwal	4.345	-0.285	1.305	5.097	9.251	4.906
	Lklu	3.459	-0.443	1.593	7.657	8.870	5.411
	Klac	-0.622	-0.125	1.887	4.669	7.978	8.600
	Calb	1.933	0.842	3.453	5.394	9.736	7.804
	Ylip	-3.644	1.534	3.200	2.192	3.057	6.701

Table S5. Heterologous *MLS1* promoter expression fold change (log_2 scale) from glucose to ethanol compared to endogenous *MLS1* expression fold change for each species before and after the diauxic shift.

Species	Heterologous *MLS1*<br>Ethanol – Glucose fold change<br>(from Table S3)	Endogenous *MLS1*<br>Plateau – Lag phase fold change [a]<br>(from Table S4)
S. cerevisiae	7.56	7.91
S. paradoxus	7.06	8.26
S. uvarum	6.44	5.70
C. glabrata	4.46	4.62
N. castellii	3.44	0.16
L. kluyveri	2.56	3.70
K. lactis	5.05	5.53

[a] Data for endogenous species are from Thompson *et al.* (2013). A linear regression of heterologous against endogenous *MLS1* fold changes gives an R^2 of 0.86. This regression excludes *N. castellii,* which did not appear to undergo a diauxic shift in Thompson *et al.* (2013).

Tables S6. P-values for differences in relative expression changes of *MLS1* over time compared to *S. cerevisiae* in the dynamic conditions of a switch from glucose to ethanol media.

	P-value for difference in rate of expression change between time points compared to *S. cerevisiae* [a,b]			
Species	0-15 min	15-30 min	30-60 min	60 min - acclimated
S. paradoxus	0.851578	0.738449	0.769555	0.736689
S. uvarum	0.808782	0.903919	0.721583	0.845463
C. glabrata	**3.50E-05**	0.869589	0.632714	0.372738
N. castellii	**0.035878**	0.189823	**0.02019**	0.950338
Z. rouxii	**0.030106**	**0.002798**	0.14618	0.535873
L. kluyveri	**0.000262**	**0.013679**	0.637005	0.947954
K. lactis	**0.004283**	0.366194	**0.003081**	0.051127

[a] Expression levels correspond to those shown in Figure 3A-C.

[b] The p-values shown are based on nested ANOVA where level~(timepoint/species), meaning that the expression level is a function of each species nested within each time point.

Tables S7. Significant p-values for differences in relative expression levels of *MLS1* in the dynamic conditions of a switch from glucose to ethanol media.

	P-value for difference in mean expression at each time point compared to *S. cerevisiae* [a,b]				
Species	0	15	30	60	acclimated
S. paradoxus	0.452715	0.262391	0.29149	0.367349	0.579637
S. uvarum	0.200703	0.23182	0.275625	0.16118	0.285453
C. glabrata	0.079131	**0.024743**	**0.035229**	**0.012049**	0.052946
N. castellii	**1.59E-06**	0.077563	0.539669	0.14049	0.096974
Z. rouxii	0.670524	0.166198	**7.47E-05**	**0.005469**	**0.000116**
L. kluyveri	**0.002509**	0.500891	**0.004454**	**0.014309**	**0.004374**
K. lactis	0.059916	0.2965	0.067079	0.390145	0.26356

[a] Expression levels correspond to those shown in Figure 3A-C.

[b] The p-values shown are based on nested ANOVA where level~(timepoint/species), meaning that the expression level is a function of each species nested within each time point.

Table S8. Yeast species strains used for genomic DNA.

Strain	Species	Source	Size of region used (bp)
S288C	*S. cerevisiae*	D. Botstein	825
NRRL Y-17217	*S. paradoxus*	E. Lewis	785
MCYC623	*S. uvarum*	E. Lewis	813
NRRL Y-65	*C. glabrata*	C. Kurtzman	2683
FM476	*N. castellii*	M. Johnston	1013
NRRL Y-229	*Z. rouxii*	C. Kurtzman	1030
FM479	*S. kluyveri*	M. Johnston	2315
FM423	*K. lactis*	M. Johnston	1501

File S1

List of Primers

Primers targeting yeast species genomic DNA

The following the genomic regions and primers were used for each species. For the *S. cerevisiae*, bold indicates the XmaI restriction site attached to primers. For all other species, bold indicates regions with homology to the pRS306 plasmid (forward) and the the beginning of the *S. cerevisiae MLS1* coding region (reverse).

Saccharomyces cerevisiae S288c chromosome XIV
Accession number: BK006947
Coordinates: 405533-408327 (length: 2795 bp)
Forward: ATATAT**CCCGGG**TTCTCCAATGCGTGTCGTAG
Reverse: ATATAT**CCCGGG**CCTTTGCTGAAAGCTTCAAACG

Saccharomyces paradoxus NRRL Y-17217 contig_246
Accession number: AABY01000166
Coordinates 17189-17973 (length: 785 bp)
Forward: **TCTAGAACTAGTGGATCCCCCGGG**TTTCTAATGCGAGGGTACTTTTATTTTTTTC
Reverse: **CGTTATCCAAACTGACCTTAACCAT**TTCTTTATTCTTTTATGTGCTTTACTACTTTGC

Zygosaccharomyces rouxii (strain CBS732)
Accession number CU928174
Coordinates 1321964-1322993 (length: 1030 bp)
Forward: **TCTAGAACTAGTGGATCCCCCGGG**CAGGGTTTTAACCCCGAAAAAC
Reverse: **CGTTATCCAAACTGACCTTAACCAT**GATTAACCGATAAAAATGTAATTTTC.

Naumovozyma castellii NRRL Y-12630 YM476-Contig666
Accession number AACF01000050
Coordinates 31280- 32292 (length: 1013 bp)
Forward: **TCTAGAACTAGTGGATCCCCCGGG**GGTGACCAGCTAATAAAGAATC
Reverse: **CGTTATCCAAACTGACCTTAACCAT**TGTGGTTGTGGTTGTGTTTG

Lachancea kluyveri NRRL Y-12651 chromosome E
Accession number: CM000691 AACE03000000
Coordinates: 906302-908617 (length: 2316)
Forward: **TCTAGAACTAGTGGATCCCCCGGG**TGGCTGGATATTACCAATTTTAATATTTTG
Reverse: **CGTTATCCAAACTGACCTTAACCAT**TTTTGAATATGTGAAATTGCGTCTTAG

Kluyveromyces lactis strain NRRL Y-1140 chromosome F
Accession number: CR382126
Coordinates: 2233682- 2235182 (length: 1501)
Forward: **TCTAGAACTAGTGGATCCCCCGGG**ATTTCAGTCTAATTTAAATGGTTAATGGTAC
Reverse: **CGTTATCCAAACTGACCTTAACCAT**GATGAATCTTTAATAAAGTTTTCTGATAGTAG

Saccharomyces uvarum MCYC 623 contig_517
Accession number AACA01000260
Coordinates 1689-2501 (length 813)
Forward: **CTAGAACTAGTGGATCCCCCGGG**TTTCTAATACGGAGGTACTTTTATTTTTTTTTAACC
Reverse: **CGTTATCCAAACTGACCTTAACCAT**TTTTTTCCTTGCTGTGTACTATTATTACTTG

Candida glabrata CCTCC M202019 unplaced genomic scaffold CGRCMSCAFFOLD_1
Accession number: KI545896
Coordinates: 441629-444311 (length 2683)
Forward: **TCTAGAACTAGTGGATCCCCCGGG**GTCTTCAATATATTATATATATATCTGCCTTTATC

Reverse: **CGTTATCCAAACTGACCTTAACCAT**TTTTTTAGTTTGCTATCTTTATGTTAATTTTGG

Primers targeting pRS306-ScMLS1
For Gibson Assembly (New England Biolabs) the pRS306-ScMLS1 plasmid was amplified using the primers:

Forward: **ATGGTTAAGGTCAGTTTGGATAACGTC** (start of the *S. cerevisiae MLS1* coding region)
Reverse: **CCCGGGGGATCCACTAGTTCTAG** (pRS306 plasmid region 5' of the site of insertion of the *S. cerevisiae MLS1* region)

***MLS1* promoter deletion primers**
The following primers were used to create plasmids with regions of the *S. cerevisiae MLS1* promoter deleted. These primers amplified the entire pRS306-ScMLS1 plasmids (except for regions in between the reverse and forward primers that contained the region to be deleted).

For creating a plasmid with 1 Cat8 site removed (Cat8 site closest to the promoter):
Forward: TTTTTT**AGATCT**GCGGTTGTCCCCTTTCCCGG
Reverse: TTTTTT**AGATCT**GATATGGGGATTTCCATTGAGCCG.

Both Cat8 sites deleted:
Forward: TTTTTT**AGATCT**GCGGTTGTCCCCTTTCCCGG
Reverse: TTTTTT**AGATCT**GTAGGGTAATACTGTTGCGTATATAGTG

Mig1 region deleted:
Forward: TTTTTT**AGATCT**CTACACTGGCTACCGATTTAACTCATCTTC
Reverse: TTTTTT**AGATCT**CCGGGAAAGGGGACAACCGC

Entire promoter deleted excepted the basal promoter:
Forward: TTTTTT**AGATCT**CTACACTGGCTACCGATTTAACTCATCTTC
Reverse: TTTTTT**AGATCT**GGCAAAATAACCAGGAAAAACAAATATTG
